# Protein-Protein Interactions as a Strategy towards Protein-Specific Drug Design: The Example of Ataxin-1

**DOI:** 10.1371/journal.pone.0076456

**Published:** 2013-10-14

**Authors:** Cesira de Chiara, Rajesh P. Menon, Geoff Kelly, Annalisa Pastore

**Affiliations:** 1 National Institute for Medical Research of the Medical Research Council, London, United Kingdom; 2 Medical Research Council Biomedical NMR Centre, London, United Kingdom; University of Bologna & Italian Institute of Technology, Italy

## Abstract

A main challenge for structural biologists is to understand the mechanisms that discriminate between molecular interactions and determine function. Here, we show how partner recognition of the AXH domain of the transcriptional co-regulator ataxin-1 is fine-tuned by a subtle balance between self- and hetero-associations. Ataxin-1 is the protein responsible for the hereditary spinocerebellar ataxia type 1, a disease linked to protein aggregation and transcriptional dysregulation. Expansion of a polyglutamine tract is essential for ataxin-1 aggregation, but the sequence-wise distant AXH domain plays an important aggravating role in the process. The AXH domain is also a key element for non-aberrant function as it intervenes in interactions with multiple protein partners. Previous data have shown that AXH is dimeric in solution and forms a dimer of dimers when crystallized. By solving the structure of a complex of AXH with a peptide from the interacting transcriptional repressor CIC, we show that the dimer interface of AXH is displaced by the new interaction and that, when blocked by the CIC peptide AXH aggregation and misfolding are impaired. This is a unique example in which palindromic self- and hetero-interactions within a sequence with chameleon properties discriminate the partner. We propose a drug design strategy for the treatment of SCA1 that is based on the information gained from the AXH/CIC complex.

## Introduction

Ataxin-1 (ATX1) is the protein responsible for the currently incurable spinocerebellar ataxia type 1 (SCA1), a dominant neurodegenerative misfolding disease characterised by ataxia, progressive motor deterioration [1], [2] and degeneration of selected neuronal subtypes [3]. ATX1 is part of a larger family of diseases that includes Huntingtin and other spinocerebellar ataxias. They are all believed to be caused by expansion of a polymorphic CAG repeat tract in the coding region of the respective genes [1]. However, for an increasing number of the polyQ diseases it has been shown that regions outside the polyQ tract profoundly modify and independently contribute to protein aggregation [4], [5], [6], [7], [8], [9].

The pathogenic mechanism of SCA1 seems particularly complex. PolyQ expansion above a threshold of ∼39 repeats is essential for disease development as shown by the linear correlation between the length of the longer uninterrupted polyQ tract and the age at onset [1]. However, overexpression of non-expanded ATX1 (30Q) in flies and mice causes phenotypes similar to those caused by overexpression of drosophila ATX1 which lacks the polyQ tract but different from those observed for polyQ peptides [10], [11] strongly suggesting the presence of at least a second aggregation hotspot. This has been identified in the AXH domain (SMART SM00536) [12], a motif responsible for transcriptional repression and RNA-binding activity of ATX1 [5], [11], [13], [14], [15], [16], [17], [18]. The AXH domain is also necessary and sufficient for the majority of the known ATX1 interactions with other proteins, most of which are transcriptional regulators (SMRT, Gfi-1, CIC, Sp1 and Tip60) [11], [13], [14], [15], [16].

Although AXH does not contain a polyQ tract and is sequence-wise distant from it, independent evidence indicates an important role of AXH in ATX1 aggregation. We have recently demonstrated that the AXH domain has an unusual chameleon oligonucleotide-binding (OB) fold, a structural motif involved in nucleic acid and protein recognition [19]. In solution, the isolated AXH forms a complex equilibrium between monomer, dimer, tetramer and higher molecular weight species that are on-pathway to protein misfolding and fiber formation [20]. The presence of the AXH domain favours formation of intra-nuclear aggregates of expanded ATX1 in eukaryotic cells [5]. The evidence of an involvement of AXH as an independent aggregation hotspot is so compelling that Zohgbi and coworkers went as far as suggesting that ‘the AXH domain but not the expanded polyQ tract is necessary to generate the ATX1 gain-of-function phenotype in flies or mice’ [11].

In our quest for a treatment of SCA1, we reasoned that protein-protein interactions could be the basis of a strategy to prevent ATX1 aggregation in a specific and effective way. This concept is supported by several examples which indicate protein-protein interaction as one of the way proteins protect themselves from aberrant aggregation [21]. If we could exploit this concept, we might be able to obtain protecting molecules that engage in specific interactions with the protein of interest providing an approach different from the use of small compounds such as polyols and phenols [22]. Although somewhat effective *in vitro*, these compounds are typically non-specific and tend to act on more than one pathway *in vivo*.

With this aim in mind, we considered the interaction between the AXH domain and a linear motif of the CIC protein, the human orthologue of the *Drosophila* transcriptional repressor capicua. CIC is an HMG-box protein that forms a stable interaction both with wild-type and expanded ATX1 and participates in the formation of large native complexes in mouse cerebellum [23]. CIC is the only one of the ATX1 binding partners whose expression levels are significantly reduced in ATX1-null mice [14] and whose structure in a complex with ATX1 is known [24]. Reduction of the CIC levels correlates with mitigation of the SCA1 disease phenotypes [25]. It was recently shown that a peptide 28 amino acids-long spanning the N-terminus of CIC competes dimer formation of AXH but in turn causes CIC mediated dimerisation [24].

Here, we show how a ten residues peptide is the region necessary and sufficient to bind to ATX1 AXH in solution and out-compete dimerisation and further aggregation by stabilizing the AXH monomer. Recognition occurs with high affinity but low sequence specificity, suggesting that the mechanism is not just restricted to CIC but is a more general mode of interaction of ATX1 with protein partners. The structure of the complex describes, at a molecular level, the mechanism by which ATX1 exerts its transcription co-repressor function and constitutes a novel example of a conformational switch. Our results provide the basis for the development of a new strategy for the specific treatment of SCA1 based on peptide mimetics of the CIC peptide.

## Results

### Defining the Sequence of the CIC Linear Motif Recognized by AXH

**Figure 1:**
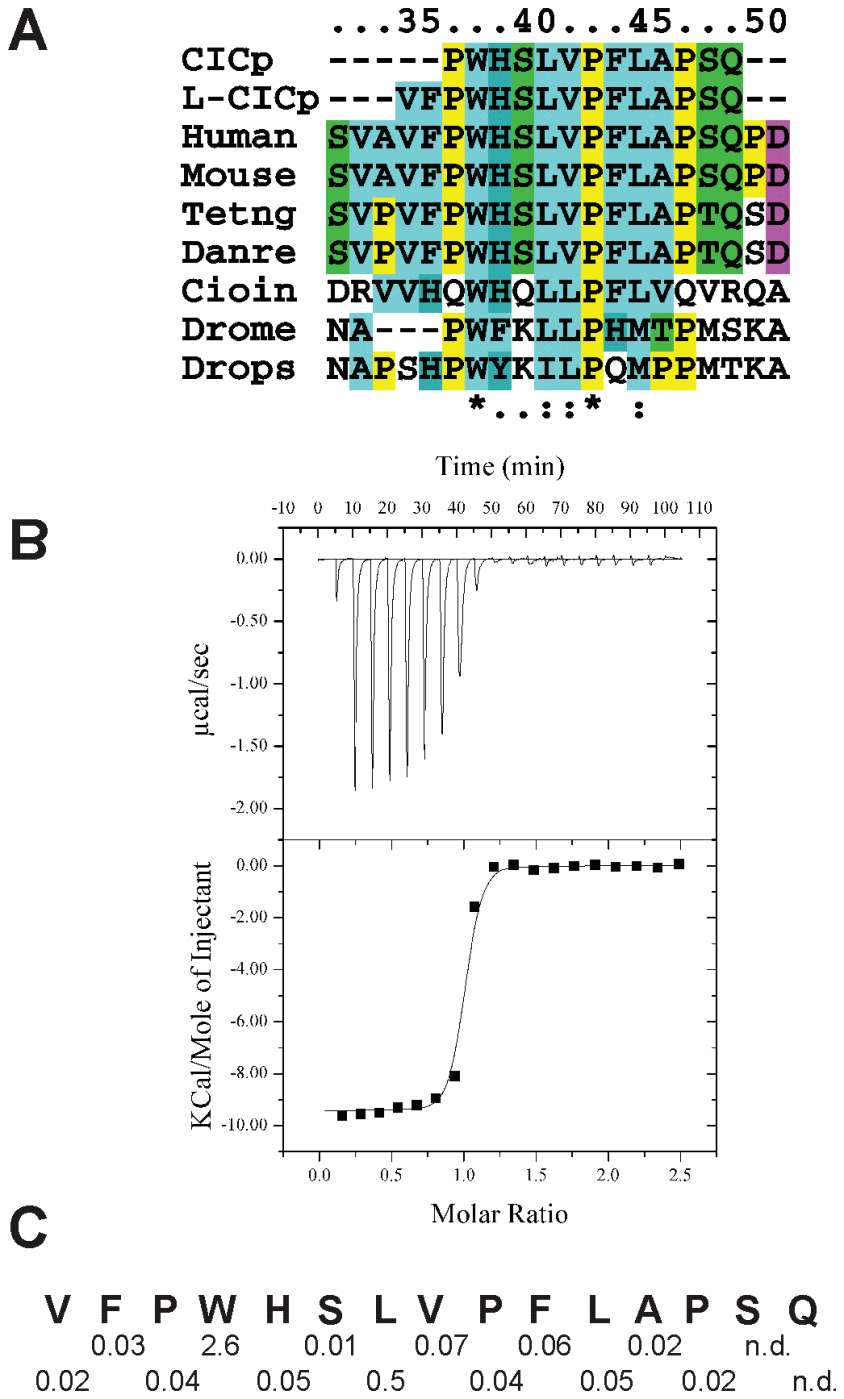
Interaction of the CIC peptides with ATX1 AXH. A) Sequence alignment of CIC sequences from different species. Numbering of CIC is according to UniProtKB/Swiss-Prot Q96RK0. The ATX1 interacting motif spanned by L-CICp sequence is also present in the Capicua-like protein/double homeodomain 4 fusion protein (CIC/DUX4 fusion) involved in the up-regulation of PEA3 family genes in Ewing-like sarcomas [48]. B) ITC profile for the binding of ATX1 AXH to L-CICp. Row calorimetric data (upper plot) and resulting integrated enthalpy data fit to a single-site binding model (lower plot). C) Dissociation constants (µM) of ATX1 AXH to wild-type and mutant L-CICp peptides where each position was individually mutated to alanine with the exception of A45 which was mutated to glycine. For comparison, the Kd of wild-type AXH with L-CICp is 0.03 µM.

We first checked the minimal length of CIC that would retain a high affinity interaction without causing further CIC-mediated dimerisation. We thus designed the peptide by selecting the region conserved among species (WHSLVPFL) (**Figure 1A**). Flanking residues from the natural sequence were added at the N- and C-termini (one and four residues respectively) to increase solubility (CICp). Isothermal titration calorimetry (ITC) measurements showed that the AXH domain interacts with CICp with a dissociation constant (Kd) of 8 µM. The addition of only two amino acids, V34 and F35, at the N-terminus (L-CICp) significantly increases the peptide affinity as the Kd decreases by two orders of magnitude (∼33 nM) (**Figure 1B**). Interestingly, the two additional amino acids are fully conserved throughout vertebrates, but not in *D. melanogaster*, even though interaction between CIC and ATX1 orthologues has also been described in these organisms [14]. Further N-terminal extension to include up to four additional amino acids resulted in a peptide with a Kd comparable to that of L-CICp (∼24 nM).

To assess the specific contribution of each amino acid in the peptide, we individually mutated to alanines the residues from V34 to P46 with the exception of A45, which was mutated to a glycine. Only mutation of residues W37 and L40 affects binding with a decrease of the Kd of 100- and 10-fold respectively, whereas all the other mutations did not significantly influenced the affinity (**Figure 1C**).

These results confirm and quantify the affinity of the interaction at the amino acid level. They also show that the presence rather than the specific individual nature of extra N-terminal amino acids is important to stabilize the complex.

### The AXH Domain Binds the CIC Linear Motif as a Monomer

**Figure 2:**
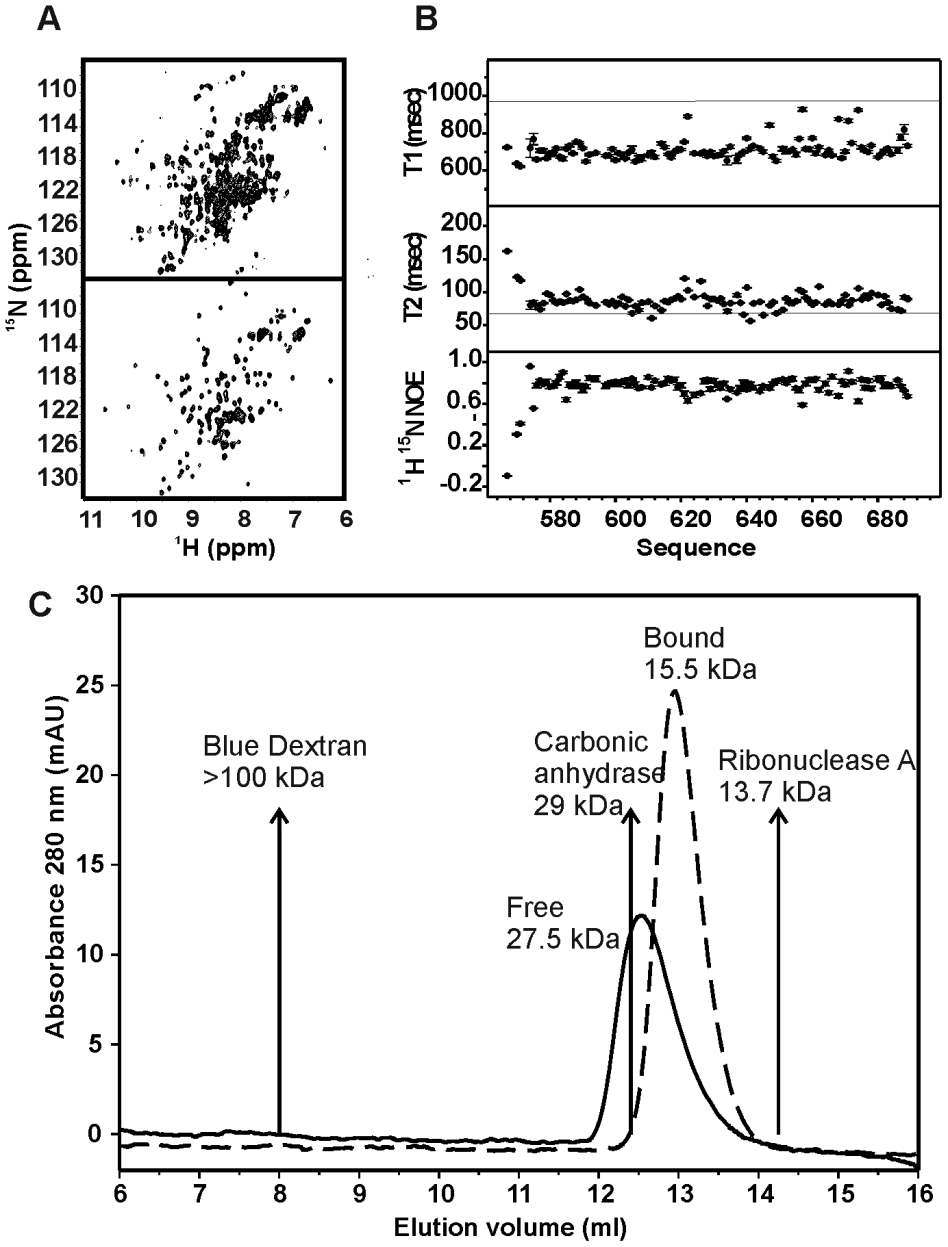
The ATX1 AXH becomes monomeric in the complex with L-CICp. A) [^15^N-^1^H]-HSQC NMR spectra recorded at 27°C of AXH for the free form (top) and the complex with L-CICp (bottom) (AXH:L-CICp molar ratio 1∶2). B) T_1_, T_2_ and ^15^N-^1^H NOE plots for AXH in complex with L-CICp as measured at 27°C and 600 MHz. It is clear from these plots that the AXH structure is overall rigid with the exception of the AXH N-terminal tail and sparse residues located in short loop regions. Horizontal straight lines indicate the equivalent average values observed in isolated AXH [20]. C) Size exclusion chromatography study. The elution profiles of isolated (Free) and complexed (Bound) AXH domain are compared with the indicated markers.

The [^15^N^1^H]-HSQC spectrum of the isolated ATX1 AXH contains more resonances than expected on the basis of the sequence with the peak intensities being highly heterogeneous (**Figure 2A** **, top**). Using a combination of NMR, analytical ultracentrifugation, X-ray and small angle scattering, we have previously shown that the observed behaviour is consistent with the presence of a monomer-dimer-tetramer equilibrium over a wide range of concentrations as well as with conformational heterogeneity of the different protomers [20]. When we titrated the ^15^N-labelled AXH with unlabelled L-CICp, the NMR spectrum underwent a dramatic change (**Figure 2A** **, bottom**). The spectrum of AXH fully saturated with L-CICp (at a ca. 2∶1 L-CICp:AXH molar ratio) is indicative of the presence of a single species (i.e. a monomer or a symmetric multimer) as it contains the expected total number of resonances.

The NMR relaxation parameters reveal well the state of aggregation of the domain. At the same field (600 MHz) and temperature (27°C), we observe a drop of the average T_1_ values from 1s to 700 ms and an increase of the average T_2_ values from 70 ms to 80 ms (**Figure 2B**, to be compared with values previously published for the isolated AXH [26]). From these parameters, we estimated a correlation time (τc) of 6.5 ns for the complex, a value consistent with a monomeric species of the molecular weight of AXH. This value should be compared to the τc of 12 ns obtained for the AXH dimer before addition of the peptide. This evidence was further confirmed by size exclusion chromatography measurements (**Figure 2C**): while isolated AXH elutes around 12.5 mL, which is close to the carbonic anhydrase standard (29 kDa), the complex elutes at a volume compatible with a 15.5 kDa species, which is approximately the size of the AXH monomer bound to L-CICp.

These results indicate that interaction with the peptide out-competes dimer formation by affecting directly or indirectly the dimer interface and leads to a complex of the monomeric AXH with CIC.

### L-CICp forms a Tight Complex with AXH

**Figure 3:**
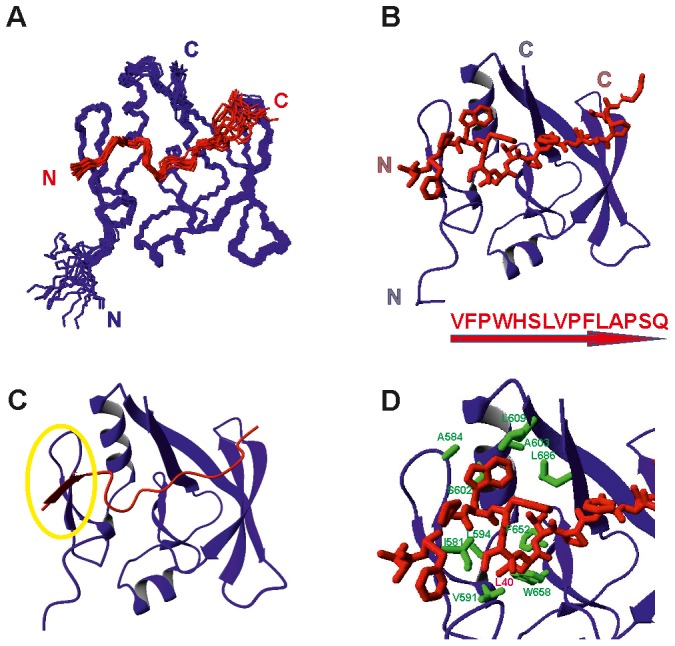
Solution structure of the ATX1 AXH/L-CICp complex. A) Structure bundle of the lowest energy 15 structures of the complex superimposed on residues 575–689 of the AXH domain. The backbone of the domain is shown in blue, L-CICp in red. B) Ribbon representation of AXH with the peptide shown in a full heavy atom representation. The N- and C-termini are marked. The peptide sequence is indicated at the bottom together with the direction N-to-C indicated by an arrow. C) The same as in B) but showing the peptide as a ribbon. The backbone atoms of V34 and F35 pair with residues of the domain and form an additional short β-sheet (highlighted with a yellow circle). D) Details of the most important interactions of the peptide with the domain. The side chains of AXH residues that form extensive interactions with L-CICp W37 and L40 are shown explicitly.

To gain more insights into the recognition of ATX1 by CIC in solution that could then be compared with the crystal structure of a complex with a longer CIC peptide [24], we solved the structure of the complex of the AXH domain with L-CICp based on intra- and inter-molecular distance restraints. The bundle of solutions is well defined, with the energetically best 15 structures superimposing with a root mean square deviation (r.m.s.d.) of 0.65±0.14 Å on backbone atoms of the structured regions (L-CICp 34–44, AXH 575–689) (**Figure 3A,B** **and Table S1**). The peptide conformation and its interaction with the domain rely on 144 restraints, 100 of which being unambiguous.

The peptide binds in an extended conformation. Overall the backbone and side-chains of L-CICp from V34 to L44 are well defined. The r.m.s.d steadily increases from A45 to Q48 as compared to the preceding residues, thus indicating that the last four amino acids experience significantly higher flexibility and/or conformational disorder. This agrees with the observation that residues P46-Q48 are not observed in either the X-ray structures of AXH-CIC_21_ (4J2J) or AXH-CIC_28_ (4J2L) [24]. Assignment of the L-CICp NMR resonances in the bound form also reveals the presence of a double set of resonances for the residues A45, P46, S47 and Q48 suggesting the presence of a *cis-trans* isomerization equilibrium at P46, with a prevalence of the *trans* form.

Interestingly, the two N-terminal amino acids of the peptide, V34 and F35 that are non-conserved between vertebrates and *Drosophila* but confer a two-order magnitude tighter affinity to the binding, make main-chain–main-chain contacts with the protein and form a short β-strand that pairs up in a parallel mode with I580 and I581 of the AXH β1-strand (**Figure 3C**). This explains why the N-terminally longer peptide L-CICp binds more tightly than its shorter version CICp with low specificity for the specific nature of the individual side-chains of residues 34 and 35. As proven by the alanine scan (**Figure 1C**), it is the elongation of the peptide rather than the specific side-chain identity of these residues that is important for the formation of this extra stabilizing structural element.

Besides these contacts, the L-CICp side-chains form extensive contacts with conserved residues of the AXH core: V34 (I580, L588), F35 (P573), W37 (F599, S602, L609, L686), L40 (I581, V591, L594), V41 (L686) and L44 (V641, V643). Most of the interactions between the protein and the peptide are mediated by the main- and side-chain of W37 and by the L40 and L44 side-chains (**Figure 3D**), as directly supported by intermolecular NOEs derived from NMR filtered experiments. The structure explains why W37 plays a major role in determining the high affinity between the peptide and the AXH domain: the W37 side chain inserts itself into a groove formed by A584 of the turn between β1 and β2, S602 of helix1, L609 of β3 and L686 of β9. ITC analysis of the alanine mutants shows that among the remaining conserved large hydrophobic residues of the peptide that orient their chains towards the core of the domain (i.e. L40, L44 and V45), only L40, besides W37, appreciably decreases the affinity when singly mutated into alanine. Indeed, the side-chain of L40 is inserted into a pocket of the AXH core even more deeply than W37, and occupies a cavity defined by I581 of β1, V591 and L594, F599 of helix1, and F652, W658 and L672 of β6, β7 and N-terminus of β8, respectively. It is only apparently surprising that most of the other mutants, particularly the ones like L44 and V45 which orient their side-chains into AXH hydrophobic pockets, bind with Kds comparable to wild-type L-CICp. This observation supports the ‘plasticity’ of the AXH domain as exemplified by the mutual adaptation of the two structurally different N-termini of the protomers in the dimer [27]. The alanine scan thus indicates that small structural adjustments are allowed, and must be occurring, in the AXH core in order to adapt structurally and allow accommodation of an alanine in a pocket where bulkier side-chains, like L44 and V45, have evolved to bind, without this quantitatively affecting the global affinity of the peptide. Further data using double and/or multiple mutants of the peptide, also with amino acids other than alanine, will be required to test the limits of this model and gain more information on amino acids which could be allowed or disallowed in key positions of the L-CICp sequence. Confirmation of the generous structure adaptability of the domain comes also from mutations tested on the protein side [24]. The authors could detect a decrease in the affinity between AXH and peptide CIC_21_ only with combined mutations of highly conserved residues, in pairs or with a triple mutant, whereas single mutations showed affinity comparable to the wild-type [24].

Combining the analysis of the solution structure with our ITC results on the shorter CICp peptide we can thus argue that the stretch P36 to L44 represents the minimal region of CIC that is necessary and sufficient to disrupt AXH homodimerization and form a monomeric complex.

### L-CICp Binds to the AXH Surface Involved in Dimerisation of the Domain

**Figure 4:**
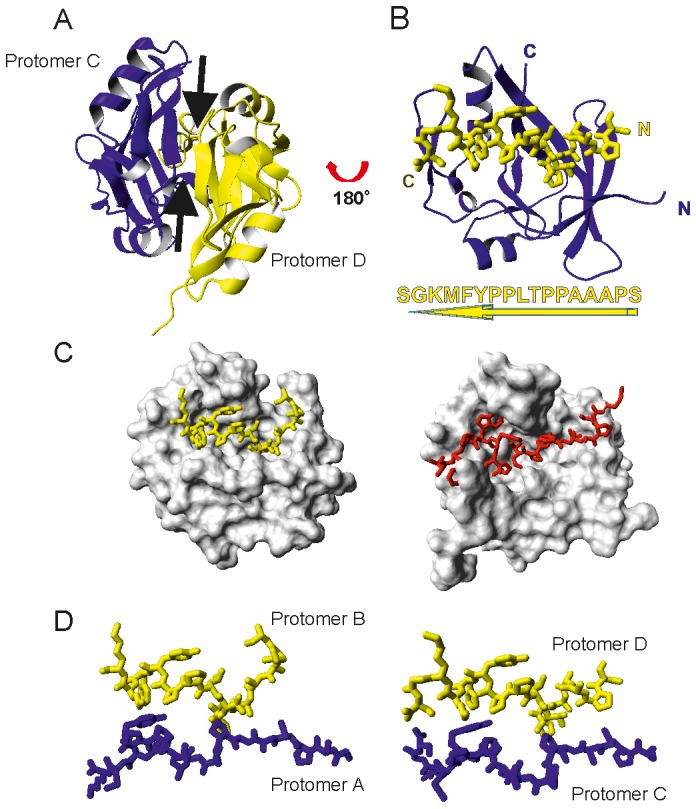
L-CIC sits in the same cavity that forms the ATX1 AXH dimer interface. A) The AXH dimer (C and D chains from the X-ray structure 1OA8). The dimer interface is dominated by the N-termini of the two chains (indicated with two arrows). The two N-termini pack against each other in a parallel orientation. B) As in A) but rotated by 90 degree around the y axis. For clarity most of chain D is omitted except for the N-terminus (in yellow) which packs against the same cavity occupied by L-CICp (compare with Figure 3). The sequence of the N-terminus (residues 563–579) is indicated at the bottom together with the direction N-to-C indicated by an arrow. Notice that the orientation is opposite to that occupied by the CIC peptide. C) Comparison of the AXH cavity (chain A) which hosts the N-terminus of the other protomer in the dimer (left, the N-terminus of chain B is shown in yellow) and L-CICp in the complex (right, the peptide shown in red). The orientation is the same as in Figure 3 and 4B. D) Zoom on the four N-termini to highlight the asymmetry of the interfaces.

Comparison between the AXH dimer and the CIC complex reveals interesting features. The fold of the AXH monomer in the complex is similar to that of the individual protomers in the free ATX1 AXH (**Figure 4A**). The average structure, as calculated by Wheatsheaf [28], shows average r.m.s.d. values of 1.9±0.4 Å and 2.5±0.4 Å respectively for the backbone and heavy atoms of residues 575–688 when superimposed to chains A, C and D of the crystal structure 1OA8 [27]. The more divergent chain B superposes similarly well when excluding the loop between residues 596–610 due to the extensive unfolding of helix 1. In the individual dimers, the protomers pack against each other as two right hand palms that touch each other in an antiparallel fashion so that the two thumbs are close in space and parallel. Accordingly, the first seven N-terminal amino acids (A567–P573, corresponding to the thumbs) of each monomer pack against the core of the other protomer (the palm of the other hand) and are extensively involved in defining the dimer interface (**Figure 4B**).

In the complex, the peptide replaces, in a reversed orientation, the N-terminus of the other dimer protomer, having a similar effect in shielding the hydrophobic core of the AXH domain from solvent exposure (compare **Figure 3B** **and** **4B**). The peptide pushes out the N-terminal residues of the domain (A567–P573) which, in the complex, are unstructured and flexible and do not make contacts with the rest of the domain or with the bound peptide. In our hands metaphor, they are like a thumb in a fully open hand. What is particularly interesting is that L-CICp does not simply replace the dimer interface but completely remodels it (**Figure 4C**). In the dimer, the cavity in the concave region of the approximately sickle-cell-like shape of the AXH monomer accommodates the N-termini of both protomers in a parallel orientation. The peptide replaces both termini and gains a completely different arrangement mediated by the side-chains of W37 and L40. These bulky hydrophobic interactions are absent in the N-terminus sequence, which is dominated by prolines. Likewise, despite sharing the same cavity, the backbone conformations of the peptide in the complex and of the N-termini in the dimer are quite different as are also the contacts between the side-chains with the surroundings, suggesting very distinct structural solutions.

An unusually strong adaptability of the AXH domain to the specific partner is also highlighted by the asymmetry that the protomer structures have in the dimers, especially at the N-termini, which adopt different conformations in each protomer (**Figure 4D**).

These results indicate a direct competition between protein-protein interactions and self-association of ATX1 AXH and confirm the importance of the extraordinary structural plasticity of the domain in setting up the rules for protein-protein recognition.

Partner recognition plays an essential role in determining the state of aggregation of ATX1.

### Interaction with L-CIC Stabilizes the AXH Monomer and Prevents Aggregation

**Figure 5:**
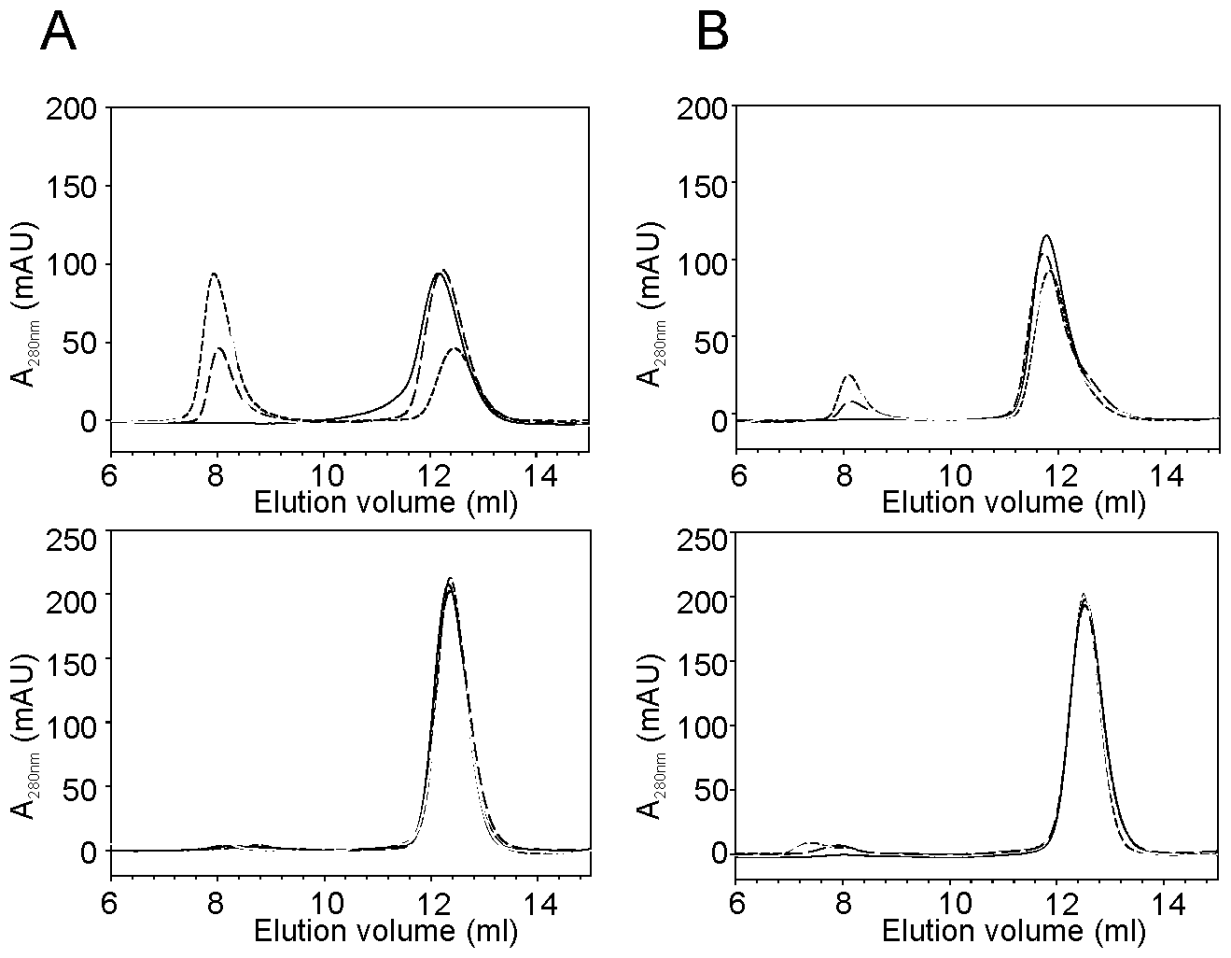
AXH aggregation monitored by size exclusion chromatography. Analytical gel-filtration chromatography of AXH_568–694_ and AXH_567–689_. **A**) Elution profile for a 60 µM AXH_568–694_ sample incubated at 37°C in absence (top panel) and presence (bottom panel) of L-CICp (1∶3 AXH:L-CICp). Top panel: Black solid line, fresh sample; long dash, short dash and grey solid line after 1, 4 and 7 days of incubation at 37°C. Bottom panel: Black solid line, fresh sample; long dash, short dash and grey after 1, 4 and 30 days of incubation at 37°C, respectively. **B**) Elution profile for a 60 µM AXH_567-689_ sample incubated at 37°C in absence (top panel) and presence (bottom panel) of L-CICp (1∶3 AXH:L-CICp). Top and bottom panels: Black solid line, fresh sample; long dash, short dash, and grey solid line after 1, 8 and 14 days of incubation at 37°C, respectively.

We had previously demonstrated that the AXH has an intrinsic tendency to aggregate and form fibers in vitro and is able to modulate the aggregation of the full-length expanded ATX1 in vivo [5], [29]. We wondered whether, given its properties, L-CIC could also out-compete aggregation and misfolding of the AXH domain. We tested in parallel two different constructs of the AXH domain, one spanning residues (AXH_568–694_) used in the previous characterization [5], [29] and a second C-terminally shorter construct (AXH_567–689_) used for structure determination of the L-CICp complex. Both constructs were incubated at 37°C (assumed as a reference for the physiological temperature) in the absence and presence of L-CICp (in 3 equivalent molar excesses to AXH). Aggregation was monitored over time by analytical size-exclusion chromatography. Both constructs aggregated in their free forms resulting in a decrease of the initial peak of the ‘monodispersed’ species and the appearance over time of a second species which ran with the void volume of the Sephadex 75 column (MW ≥100 kDa) (**Figure 5**). AXH_568-694_ showed faster kinetic than AXH_567–689_: after 8 days of incubation, AXH_568–694_ was 100% aggregated, whereas the AXH_567–689_ sample still showed ∼60% of the initial species after 14 days. This observation is in line with what previously observed, i.e. that C-terminal elongation accelerates aggregation [18]. For both constructs, complex formation with L-CICp completely hampers aggregation: AXH_568–694_/L-CICp complex remains unchanged after 30 days at 37°C.

These results clearly demonstrate that interaction with the peptide is effective in stabilizing the monomeric species and preventing aggregation of the domain.

## Discussion

Protein-protein interactions play an essential role in determining the localization, functions and fate of proteins in the living cell. A main challenge for Structural Biology is to understand the rules that control interaction specificity, a topic of particular importance, for instance, in interpreting how proteins discriminate between functional interactions and self-assembly and aggregation. The complexity of this task arises not only from the number of proteins expressed by the organism (which, taking into account alternative splicing or post-translational modifications, is much higher than the number of coding genes), but also from the combinatorial cross-talk between them [30], [31], [32]. Here, we have described how the same protein domain can discriminate between self-assembly and other partners.

The AXH domain of the transcriptional co-regulator ATX1 has been a continuous source of surprise from the structural point of view. It is a unique example of a chameleon protein that is able to adopt distinct conformations in a way independent of mutations [27], [29], [33]. In solution, the AXH domain is able to form dimers, tetramers and larger species that interconvert with Kds in the micromolar to millimolar range [20]. We have now identified yet another aspect of the structural plasticity of the AXH domain. We have shown how the same surface of the ATX1 AXH domain can host palindromic interactions that determine partner recognition specificity and how the domain is able to modify its association state upon interaction with a cellular partner. A key role in these interactions is played by the AXH domain N-terminus. In the dimer, the two parallel N-termini pack against each other and form a highly asymmetric inter-molecular interface [20], [27]. In the complex, the CIC peptide displaces self-association by forming a tight interaction with nanomolar affinity and sits in the core of the sickle-cell-like OB-fold motif in an extended conformation that has reversed main-chain orientation from that observed in the dimer. This unexpected result could not have been anticipated from sequence homology as the CIC recognition motif shares with the AXH N-terminal sequence only a very vague palindromic similarity. While we were in the process of submitting the present work, the crystal structure of a complex between CIC and AXH was published [24]. The CIC construct differs from ours in being longer (21 residues from Glu28 to Gln48). While the recognition motif on the AXH is in excellent agreement with our structure in solution, binding of the longer CIC construct allows another form of dimerisation which is mediated by CIC, showing yet another lever of the intricacy of protein complex formation.

Our data have important implications for understanding both normal and pathologic functions of ATX1.

**Figure 6:**
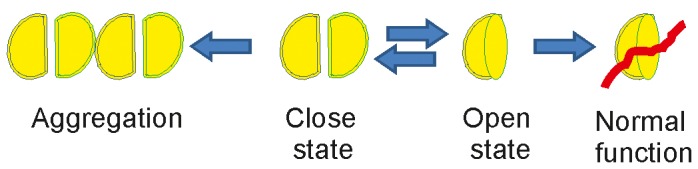
Model of the competition between protein-protein interactions and self-assembly determines the fate of the domain. In solution, the AXH monomer is in equilibrium with the dimer. In the presence of CIC, the equilibrium shifts towards the monomer that is drastically stabilized. In the absence of CIC or other cellular partners, AXH will be in a close state that is transcriptionally inactive. When this occurs, other modifiers such as polyQ expansion and/or any other perturbations will favour self-association of the domain.

AXH is an important functional domain of ATX1 already known to participate in vivo in protein-protein interactions with other partners [34]. The involvement of ATX1 in transcriptional regulation was originally inferred from the identification of several transcriptional co*-*regulators as modulators of the ATX1-mediated eye phenotype in *Drosophila* [10]. Thereafter, a number of different factors involved in transcription were discovered to interact directly with ATX1 [11], [13], [14], [15], [16], [35], [36]. The isolated AXH domain itself was found to repress transcription when tethered to DNA [5], similarly to the full-length protein [37]. However, no direct cross-linking of AXH to DNA was detected, suggesting that the interaction with other DNA-binding proteins could be required [5]. One of such proteins is CIC which was shown to require the AXH domain for interaction [11], [13], [14], [15], [16]. The structure of the CIC/AXH complex provides the structural determinants of the recognition between these proteins and a mechanistic model of how ATX1 could act synergistically with CIC to regulate transcription. The dimeric structure of free AXH can be regarded as a ‘closed’ transcriptionally inactive form (or an auto-inhibited resting state), whereas the complex is an ‘open’ functionally competent state (**Figure 6**). The unusual pseudo-barrel of AXH uses its concave side to recognize CIC, leaving available the putative RNA-binding surface for further functional interactions [27], [38]. It could be speculated that the two structural states might be related to different functional aspects of the protein, which has been proposed to play a ‘dual’ role in regulation of transcription and RNA-processing and/or metabolism [23], [39], [40]. The same recognition code could also be adopted with other ATX1 partners. Our data show in fact that, despite the high affinity, only W37 and L40 provide a significant contribution to specificity. The low homology between L-CICp and the displaced AXH N-terminal sequences together with the ability of the domain to recognize sequences with both direct and reversed backbone orientation strongly suggest that, as for the SH3 motif in proteins, this could thus be a more general mechanism to modulate the functions of ATX1. At variance with SH3 domains is, however, the plasticity of the backbone recognition: while having low sequence specificity, all SH3 complexes share a very similar backbone conformation. The AXH domain seems instead to be much more tolerant against both sequence and structural restraints according to its chameleon properties and to recognize the partner after remodelling of the binding surface.

Even more important are the implications of our findings for AXT1 self-association and therefore, potentially, for the SCA1 disease. We have previously demonstrated that the AXH dimer is on the pathway to the formation of tetramers and other higher molecular weight species as the surface of dimerisation seems to involve elements from both protomers of the dimer [20]. This implies that interaction with CIC is in direct competition with AXH self-association (**Figure 6**), in agreement with the observation that transcriptional dysregulation occurs already at the very early stages of the disease [10], [41], [42]. The existence of a competition between normal and pathological functions is an important emerging concept observed also in other diseases. We previously demonstrated for ataxin-3, another member of the polyQ family, that aggregation is the ‘dark side’ of normal function [6], [21]: the binding site of this protein for ubiquitin coincides with aggregation-prone hydrophobic surfaces that have evolved to allow partner recognition. While enforcing the concept and the importance of studying the non-pathological function of aggregation-prone proteins, our new results directly suggest a strategy for preventing AXH aggregation: it will be interesting to use the CIC peptide as a template to design peptide-mimetic molecules and to test whether and how much these lead compounds affect the overall ATX1 aggregation both by themselves and in combination with polyQ aggregation blockers. This route could be the first step towards the development of a specific strategy for the treatment of SCA1 and more in general of other polyQ diseases. Taken together, the ITC results suggest that an indole and an isobutyl group appropriately spaced from each other could mimic the W37 and L40 side-chains. They can be expected to be a strict starting requirement for the design of any molecule that can be recognised by the AXH domain and compete for interaction with CIC. Results from a thorough study of the structural-activity relationship based on the identification of disallowed mutations of the peptide, and complemented by in cell assays, will pave the way to the design of potential inhibitors of such interaction. Fine tuning of the affinity of the mimetic molecule for AXH will be required in order to appropriately modulate its interaction with CIC without significantly interfering *in vivo* with other specific functional interactions mediated by the same AXH surface. More work is therefore needed to fully elucidate the cellular functions of the AXH domain and its exact role in aggregation and to explore the implications of these findings for therapy.

## Materials and Methods

### Protein Expression and Purification

Recombinant ATX1 AXH (residues A567–K689 of human ATX1) was over-expressed in the *E.coli* host strain BL21(DE3)pLysS in Luria broth using a kanamycin-resistant pETM30 vector with Tobacco Etch Virus (TEV)-cleavable N-terminal His6-GST tag. Isotopically ^15^N- and ^13^C/^15^N labelled samples were expressed in minimal (M9) medium supplemented with ^15^N-ammonium sulphate and ^13^C-glucose as the sole sources of nitrogen and carbon respectively. The cells were grown at 37°C until an optical density (OD) of 0.6 at 600 nm was reached, before inducing protein expression with IPTG (0.25 mM) for three hours followed by harvesting. A standard purification protocol was performed using a Ni-NTA agarose column (Qiagen). The protein was further purified by FPLC size exclusion chromatography using a prepacked HiLoad 16/60 Superdex™ 75 prep.grade column (Pharmacia). Protein identity was checked by ESI mass spectrometry.

Synthetic lyophilized CIC peptides (purity >95%) were purchased from Pepceuticals Limited (Nottingham-UK), dissolved in MilliQ water that was then exchanged with the final buffer using a PD MidiTrap G10 (GE Healthcare). AXH and CIC peptides concentrations were checked by measuring UV absorbance at 280 nm using a calculated extension coefficient of 8480 and 5500 M^−1^cm^−1^, respectively.

### NMR Spectroscopy

NMR spectra for structural determination were acquired on samples containing ^15^N- or ^15^N,^13^C-labelled ATX1 AXH (0.5 mM) and unlabelled L-CICp (0.6 mM) (AXH:L-CICp molar ratio 1∶1.2) in 20 mM Tris-HCl pH 6.85, 2 mM TCEP, 0.02% NaN_3_, 8% ^2^H_2_O. The spectra were recorded at 27°C using Varian Inova spectrometers operating at 600 and 800 MHz ^1^H frequency, the 800 MHz being equipped with a triple resonance gradient Cold-Probe, and Bruker Avance spectrometers operating at 600 and 700 MHz ^1^H frequency, both equipped with triple resonance gradient CryoProbes. Resonance assignment of the AXH/L-CICp complex was performed using a standard approach as previously described [26]. AXH intra-molecular proton distance restraints were derived from ^15^N- and ^13^C-NOESY-HSQC spectra (mixing time 100 ms). Inter-molecular NOE restraints between double-labelled AXH and unlabelled L-CICp were obtained from 3D [^15^N,^13^C]-F1-filtered, ^15^N- and ^13^C-edited NOESY spectra (mixing time 100 ms), whereas L-CICp intra-molecular NOEs from a 2D [^15^N,^13^C]-F1/F2-filtered NOESY (mixing time 100 ms) [43]. T_1_, T_2_, and ^1^H-^15^N-NOE measurements were performed at 27°C and 600 MHz by adapted standard pulse sequences. Rotational correlation times (τc) for the free and L-CICp-bound form were derived using a recently devised method (Kelly & Frenkiel, in preparation) which yields a rapid and direct estimate of the ^15^N T_1_/T_2_ ratio from inspection of a series of 1D profiles.

### Structure Determination

Automated NOESY cross-peak assignment and structure determination were performed by the ARIA 2.3 software [44] based on an almost complete assignment of the complex [26] and on a large number of intra- and inter-molecular NOEs. The ARIA input used to generate the final structures consisted of AXH intra-molecular NOE cross peaks from ^15^N- and ^13^C-NOESY-HSQC spectra, L-CICp intra-molecular NOEs from 2D [^15^N,^ 13^C]-F1/F2-filtered NOESY and inter-molecular restraints from [^15^N,^13^C]-F1-filtered, ^15^N- and ^13^C-edited NOESY along with a set of φ and ψ backbone dihedral restraints derived by TALOS+ [45]. Using this set of data the structure rapidly converged to a unique fold. In the final ARIA run, a number of 200 structures were generated in iteration 8. After refinement of the 40 lowest energy structures by molecular dynamics simulation in water, 15 structures were selected as representative of the structure of the complex and used for statistical analysis. Structure quality was evaluated with the programs PROCHECK [46]. The Ramachandran plot of the final 15 refined structures showed 87.2%, 10.9%, 1.0% and 0.9% in most favoured, additional allowed, generously allowed, and disallowed regions, respectively [47].

### Quantification of the Binding Constants

Binding affinities of AXH with CIC ligands were measured at 25°C by a MicroCal Omega VP-ITC (MicroCal Inc., Northampton, USA). Both protein and peptides buffers were 50 mM Tris-HCl pH 7, 50 mM NaCl, 2 mM TCEP. Solutions of 0.041 mM AXH in the cell were titrated by injections of a total of 283 µl of 0.410 mM peptide in the syringe in 21 aliquots. Data were processed with the software MicroCal Origin version 5.0 provided by the manufacturer. All dissociation constants were averages of three measurements.

### Analytical Size Exclusion Chromatography

Size exclusion chromatography was performed using a prepacked Superdex-75™ 10/300 GL column (Pharmacia) equilibrated with a 50 mM Tris-HCl pH 7, 50 mM NaCl, 2 mM TCEP buffer solution. Samples of 150 µl of 0.2 mM ATX1 AXH, either free or in a 1∶5 excess of L-CICp, were injected and eluted using a 0.8 ml/min flow rate. Carbonic anhydrase (29 kDa), and ribonuclease A (13.7 kDa) were used as standards for the molecular mass, whereas the Blue Dextran 2000 was used for the determination of the void volume of the column.

### Analytical Size Exclusion Chromatography for Aggregation Experiments

Size exclusion chromatography was performed using a prepacked Superdex-75™ 10/300 GL column (Pharmacia) equilibrated with a 20 mM Tris-HCl pH 7, 150 mM NaCl, 1 mM TCEP buffer solution. Aliquotes of 250 µl of 60 µM AXH_568–694_ and AXH_567–689_ in the absence or presence of L-CICp (1∶4 AXH:L-CICp), incubated in 20 mM Tris-HCl pH7, 1 mM TCEP, at 37°C were injected and eluted using a 0.8 ml/min flow rate. Carbonic anhydrase (29 kDa), and ribonuclease A (13.7 kDa) were used as standards for the molecular mass, whereas the Blue Dextran 2000 was used for the determination of the void volume of the column.

#### Protein data bank accession codes

Coordinates have been deposited under accession code 2M41.

## Supporting Information

Table S1 **NMR and refinement statistics for the ATX1 AXH/L-CICp complex.**
(DOCX)
