# Supplementary material for: Protein-Protein Interactions as a Strategy towards Protein-Specific Drug Design: The Example of Ataxin-1
Source: PLoS One. 2013 Oct 14;8(10):e76456. doi: 10.1371/journal.pone.0076456 (PMC3796545; doi:10.1371/journal.pone.0076456)
Supplement: Table S1 — NMR and refinement statistics for the ATX1 AXH/L-CICp complex. (DOCX) [file pone.0076456.s001.docx]

**Table S1: NMR and refinement statistics for the ATX1 AXH/L-CICp complex.**

|  | **ATX1 AXH** | **L-CICp** |
| --- | --- | --- |
| **NMR distance and dihedral constraints** |  |  |
| Distance restraints |  |  |
| Total NOE | 2727 | 115 |
| Unambiguous | 2121 | 100 |
| Ambiguous | 606 | 15 |
| Intra-residue^a^ | 1062 | 76 |
| Inter-residue^a^ | 1159 | 24 |
| Sequential (\|*i* – *j*\| = 1) | 439 | 17 |
| Nonsequential (\|*i* – *j*\| > 1 ) | 720 | 7 |
| Hydrogen bonds | 52 |  |
| Protein–peptide intermolecular | 144 |  |
| Unambiguous | 100 |  |
| Ambiguous | 44 |  |
| Total dihedral angle restraints^b^ |  |  |
| Protein |  |  |
| φ | 87 |  |
| ψ | 87 |  |
| **Structure statistics** |  |  |
| Violations |  |  |
| Distance constraints (>0.3 Å) | 1 |  |
| Max. distance constraint violation (Å) | 0.343 |  |
| Deviations from idealized geometry |  |  |
| Bond lengths (Å) | 0.0041±0.0003 |  |
| Bond angles (º) | 0.60±0.05 |  |
| Impropers (º) | 1.88±0.23 |  |
| Average pairwise r.m.s. deviation^c^ (Å) |  |  |
| Protein (residues 575-689) |  |  |
| Heavy atoms | 1.21±0.18 |  |
| Backbone | 0.63±0.15 |  |
| Peptide (residues 34-44) |  |  |
| Heavy atoms | 1.27±0.40 |  |
| Backbone | 0.58±0.20 |  |
| Complex (residues:L-CIC 34-44, AXH 575-689) |  |  |
| Heavy atoms | 1.23±0.19 |  |
| Backbone | 0.65±0.14 |  |
|  |  |  |
|  |  |  |

^a^ Statistics among unambiguous restraints.

^b^ TALOS-derived dihedral restraints.

^c^ Pairwise r.m.s.d. was calculated among the final 15 refined structures.
